# CHEMOTHERAPY INDUCED NAIL CHANGES

**DOI:** 10.4103/0019-5154.44804

**Published:** 2008

**Authors:** Aashima Gupta, Ankit Parakh, Anand Prakash Dubey

**Affiliations:** *From the Department of Paediatrics, Maulana Azad Medical College and Associated Hospitals, New Delhi - 110002, India*

**Keywords:** *Anticancer drug*, *chromonychia*, *leukonychia*

## Abstract

Anticancer chemotherapy is associated with a variety of nail changes. We present two children who developed different nail changes, while receiving almost similar antineoplastic drugs.

## Introduction

Anticancer chemotherapy is associated with multisystem adverse effects. Nail changes as a result of chemotherapy are asymptomatic and resolve with the cessation of this therapy.^1^ They are observed more frequently amongst darker races.^2^ We present a brief report on two children who developed nail discoloration. Both patients were receiving anticancer drugs – daunorubicin, methotrexate, etoposide and cytarabine being the common agents.

## Case Reports

### Case 1

**Fig. 1:**
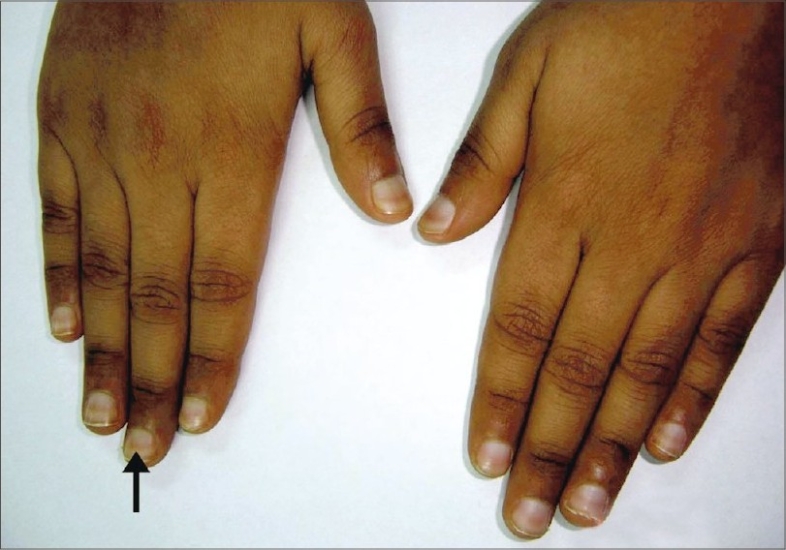
Transverse chromonychia (arrowhead) presenting as a single transverse band of red discoloration of the nail plate

An 8-year-old boy, diagnosed case of relapsed acute lymphoblastic leukemia, receiving the UK-ALL-R1 regime, developed transverse hyperpigmented bands on the nails (chromonychia) around six weeks after commencing the regime. The bands were single, transverse, 2-mm broad, spanning the entire nail breadth, red, nonblanchable, nonpalpable with smooth overlying nail surface on the nail plates of all fingers and toes. He had completed 5 cycles of chemotherapy where he had received daunorubicin, vincristine, cyclophosphamide, asparaginase, methotrexate, etoposide and cytarabine (Fig. 1).

### Case 2

**Fig. 2:**
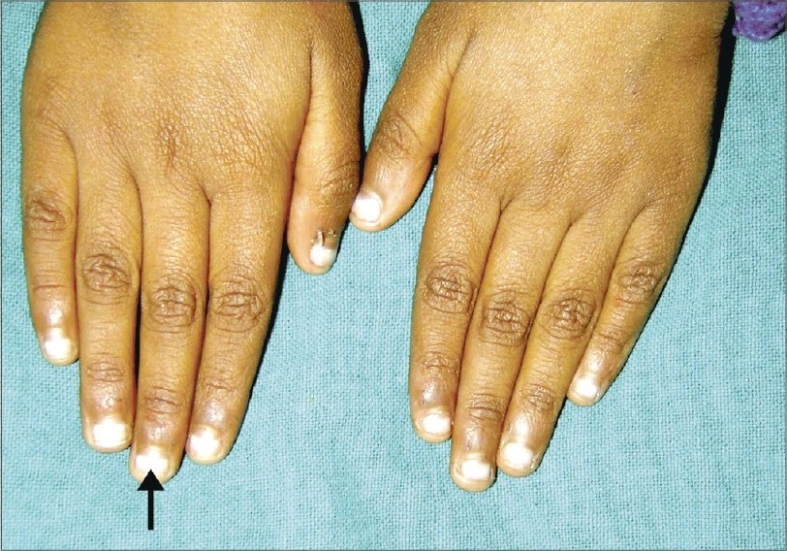
Mees' lines (arrowhead) presenting as a single transverse band of white discoloration of the nail plate

A 5-year-old boy, diagnosed case of acute lymphoblastic leukemia, receiving the UK-ALL regime developed hypopigmented transverse bands (Mee's lines) on the nail plates of all fingers and toes. This change was observed four weeks after completion of 20-week second intensification. The bands were single, transverse, 2-mm broad, spanning the entire nail breadth, white, nonblanchable and nonpalpable with smooth overlying surface. He had completed 20 cycles where he had received daunorubicin, cytarabine, methotrexate, thioguanine and etoposide (Fig. 2).

These lines were asymptomatic and moved distally with nail growth and finally disappeared.

## Discussion

Anticancer therapy is associated with varied systemic side-effects. The mucocutaneous effects can be one of the most distressing side effects to the patient, especially because of their cosmetic disadvantage.

Drug-induced nail abnormalities result from toxicity to the matrix, nail bed, periungual tissues or digital blood vessels.^1^ Chemotherapy has been associated with various types of nail changes such as nail dystrophies; different patterns of nail discoloration (known as chromonychia)^1^, leukonychia (including Mee's and Muehrcke's lines), Beau's lines, paronychia and onycholysis.^3^ Transverse bands associated with drug use generally span the entire nail breadth and are parallel to the lunula.^4^ Drugs implicated are – vincristine, hydroxyurea, etoposide, daunorubicin, bleomycin, cyclophosphamide, dacarbazine, 5-fluorouracil and methotrexate.^1^,^3^,^5^

Chromonychia induced by antineoplastic drugs has a few distinct forms. The most frequent one is melanonychia, a dark pigmentation of nails observed as diffuse, transverse, or longitudinal band patterns.^5^ The exact mechanism is still unknown, although the possibility of melanin deposition has been postulated. The patient in case one had presented with reddish transverse pigmentation bands, which is a rare form of chromonychia.

Mee's lines are single, transverse, nonblanchable bands. They result due to sudden toxic damage to the nail matrix resulting in abnormal keratinization causing altered light diffraction in retained parakeratotic onychocytes. Leukonychia was described in patients suffering from arsenic and thallium intoxication. They were also reported in various medical diseases such as myocardial infarction, acute and chronic renal failure, kidney allograft rejection, systemic lupus erythematosus, immune hemolytic anemia^6^ and Hodgkin's disease.^3^ These lines must be differentiated from Muehrcke's lines those are thicker and sometimes two lines that are transverse and parallel to lunula. Muehrcke's lines disappear on pressure. They occur due to edema in nail bed or alteration of nail plate attachment to the nail bed due to the vascular abnormalities resulting due to chemotherapy.^7^ The patient in our case report had no evidence of any metallic exposure or any other coexisting medical disease. These lines hence appear to be induced by chemotherapy.

Both leukonychia and altered pigmentation can occur due to etoposide, daunorubicin and methotrexate (all being common drugs in both cases). We thus conclude a report of two similar profile patients receiving similar anticancer drugs, but presenting with two varied forms of nail changes.

These nail changes are asymptomatic and resolve with completion of therapy; further, they do not require treatment.^1^ Synergy or an additive effect of chemotherapy agents on cellular proliferation of nail compartments is accountable for the development of this complex pattern.^5^ Early recognition is necessary to allay anxiety among patients and avoid any unnecessary work up.
